# Effects of manipulations of oblique pulling on the biomechanics of the sacroiliac joint: a cadaveric study

**DOI:** 10.1186/s12891-023-06175-6

**Published:** 2023-01-23

**Authors:** Zhun Xu, Zhiping Huang, Zhaocong Zhang, Ziyu Feng, Yiguo Yan, Qingan Zhu, Yikai Li

**Affiliations:** 1 Department of Spine Surgery, The First Affiliated Hospital，Hengyang Medical School, University of South China, Hengyang, Hunan Province China; 2 School of Traditional Chinese Medicine, Southern Medical University, Guangdong Province, Guangzhou, China; 3 Division of Spine Surgery, Department of Orthopaedics, Nanfang Hospital, Southern Medical University, Guangdong Province, Guangzhou, China

**Keywords:** Manipulations, Sacroiliac joint, Biomechanics, Stability, Cadaver study

## Abstract

**Background:**

There are many reports on the treatment of sacroiliac joint dysfunction by manipulation of oblique pulling (MOP). However, the specific mechanism of MOP on the sacroiliac joint remains unclear. This study aimed to investigate the effect of MOP on the biomechanics of the sacroiliac joint and the effect of the anterior sacroiliac ligament on the stability of the sacroiliac joint.

**Methods:**

First, MOP-F1 (F: force) and MOP-F2 were applied to nine cadaveric pelvises. Then, segmental resection of the anterior sacroiliac ligament was performed. The range of motion of the sacroiliac joint was observed in all procedures.

**Results:**

Under MOP-F1 and F2, the average total angles were 0.84° ± 0.59° and 1.52° ± 0.83°, and the displacements were 0.61 ± 0.21 mm and 0.98 ± 0.39 mm, respectively. Compared with MOP-F1, MOP-F2 caused greater rotation angles and displacements of the sacroiliac joint (*p* = 0.00 and *p* = 0.01, respectively). In addition, the rotation angles and displacements of the sacroiliac joint significantly increased after complete resection of the anterior sacroiliac ligament (*p* = 0.01 and *p* = 0.02, respectively). The increase was mainly due to the transection of the upper part of the anterior sacroiliac ligament.

**Conclusions:**

MOP-F2 caused greater rotation angles and displacements of the sacroiliac joint and was a more effective manipulation. The anterior sacroiliac ligament played an important role in maintaining the stability of the sacroiliac joint; the upper part of the anterior sacroiliac ligament contributed more to the stability of the joint than the lower part.

## Background

The sacroiliac joint (SIJ) is the largest axial joint in the human body and transmits the weight of the upper body to the lower limbs [1–3]. The SIJ is made up of a synovial part and a ligament part, so it is a diarthrodial or amphiarthrodial joint [4, 5]. Clinically, low back pain induced by SIJ disease without specific causes accounts for approximately 14.5–22.5% of cases [6]. The mechanism may include the following processes: Pathogenic factors acting on the auricular surface of the sacrum and ilium may cause injury to the ligaments or muscles around the SIJ, which can result in slight movement of the SIJ, making the joints difficult to reset. The mechanical environment of the joints may ultimately be imbalanced, and the soft tissues will be damaged. This condition is clinically referred to as SIJ dysfunction.

**Fig. 1:**
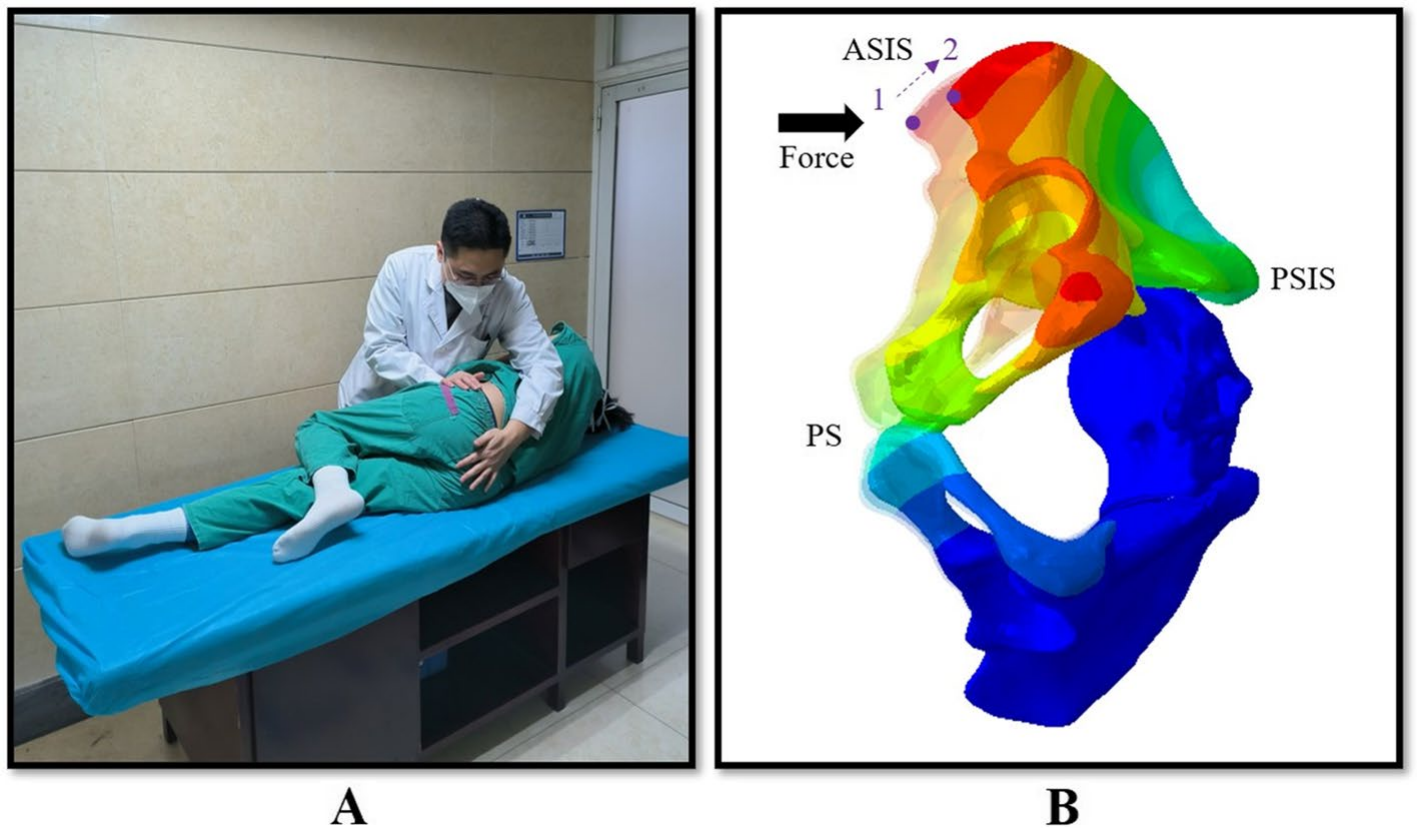
**A** showed that the therapist performed MOP on the patient. **B** showed the two positions of pelvis with and without MOP. 1: The position of ASIS without MOP, 2: The position of ASIS with MOP. MOP: Manipulation of oblique pulling. ASIS: Anterior superior iliac spine. PSIS: Posterior superior iliac spine. PS: Pubic symphysis

Manipulation is a common therapy for SIJ dysfunction [7–10]. At present, there are many clinical reports on the treatment of SIJ dysfunction by manipulation of oblique pulling (MOP). Generally, when the patients receive two manipulative treatments within a week, the pain symptoms will be significantly relieved [11–13]. The procedure of MOP is as follows: the patient is in the right decubitus position. The right lower extremity is straight, and the left lower extremity is slightly bent. The therapist stands at the patient’s ventral side. The therapist holds the patient in position with one hand on the back of the scrum and the other hand on the anterior superior iliac spine, pushing the ilium towards the back (Fig. 1). However, there are few studies about the mechanism of manipulation. Some researchers believe that MOP can cause ilium rotation and enlarge the joint space, which might be the reason for pain relief [14, 15]. However, some authors have stated that the SIJ not only had a firm wedge-shaped bone structure but also had strong ligaments around it, so it was very stable. It was difficult for manipulation to pull the dislocated SIJ back. Manipulation might relieve pain by relieving ligament spasms around the joint [16, 17]. Therefore, it is not clear whether MOP can pull back the dislocated SIJ or what the specific mechanism of MOP is. In addition, therapists often apply manipulation based on their own experience. There is no unified standard for MOP. It is also unknown whether different directions of manipulative force can produce different biomechanical effects.

The ligaments surrounding the SIJ include the anterior sacroiliac ligament (ASL), long posterior sacroiliac ligament (LPSL), short posterior sacroiliac ligament (SPSL), interosseous sacroiliac ligament (ISL), sacrospinous ligament (SS), and sacrotuberous ligament (ST). The ligaments play a vital role in maintaining the stability of the SIJ [18–21]. A finite element study found that reducing the stiffness of ligaments could significantly increase the range of motion (ROM) of the SIJ and increase the stress on the joint surface. During flexion, extension, and axial rotation of the SIJ, the strains of the ISL, ASL and SS were the largest [22]. Bohme et al. observed that the ASL and ST bore the greatest load in anteroposterior compression pelvic injuries [19]. It follows that the ASL is an important structure for pelvic stability. However, the effect of the ASL on the stability of the SIJ under MOP has not been studied.

Thus, this study aims to explore the relative displacement and rotation angle of the SIJ under MOPs in two loading directions and the effect of each part of the ASL on the stability of the SIJ based on a cadaveric biomechanical experiment.

## Methods

### Preparation of specimens

**Fig. 2:**
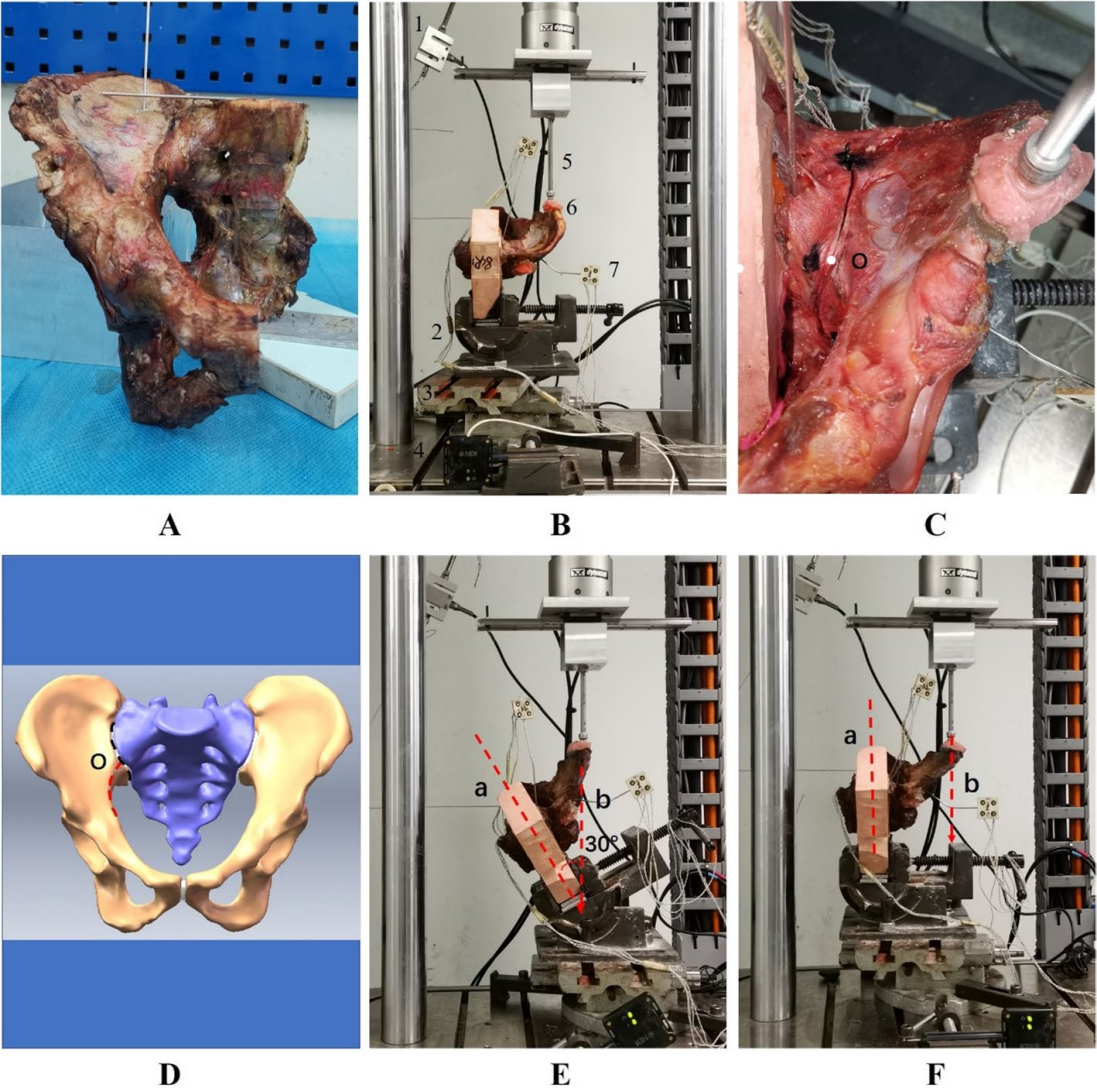
**A** showed the view of hemi-pelvic specimen. **B** showed the attachment of the pelvis to the testing machine. 1: force sensor, 2: fixture, 3: horizontal platform, 4: NDI optotrak certus, 5: loading rod, 6: loading point in ASIS, 7: marker. **C** and **D** showed the upper part and lower part of ASL. The “O” point was the intersection of the black dashed line and the red dashed line and it was the cutoff point between the upper part and lower part of ASL. The black dashed line showed the SIJ. The red dashed line showed the arcuate line. **E** and **F** showed the MOP-F1 and MOP-F2 were applied to the hemi-pelvis specimen, respectively. Line a presented sagittal plane of pelvis. Line b presented the direction of loading force. ASIS: Anterior superior iliac spine. ASL: Anterior sacroiliac ligament. MOP: Manipulation of oblique pulling

This study was approved by the Southern Medical University Medical Ethics Committee (ChiECRCT20210191). Nine fresh-frozen cadaveric hemi-pelvises (five males, four females, aged 20–60 years) were obtained from the Department of Anatomy at Southern Medical University. All trials were performed in accordance with the principles of the Declaration of Helsinki. These specimens were free of fractures, tumours, malformations, or serious osteoporosis. The skin, fat and muscle tissues were removed. Ligaments around the SIJ on one side, including the ASL, LPSL, SPSL, ISL, SS and ST, were all maintained on the specimens (Fig. 2A).

### Pelvic fixation and loading points

The middle part of the sacrum was embedded in plaster, and the hemi-pelvis was placed in a standard position, that is, the symphysis pubis and the bilateral anterior superior iliac spine were positioned in the standard sagittal plane.

The hemi-pelvis specimen with plaster embedded was fixed in the supine position on an adjustable angle fixture. The loading points were on the anterior superior iliac spine (ASIS). The soft tissue around the ASIS was removed as cleanly as possible. The screw track was preformed by an electric hand drill and strengthened by denture base resin solution. A screw (25*3.5 mm) was inserted into the ASIS through the screw track immediately, with the direction of the screw perpendicular to the sagittal plane of the pelvis. Loading point 1 was achieved. Then, the hemi-pelvis was rotated 30° along the vertical axis through fixture rotation. In the same way, a screw was inserted at the ASIS and perpendicular to the platform for the preparation of loading point 2. Finally, the denture base resin solution was used to embed the screw, and the loading point was moulded into a ball and socket shape to connect the ball head of the loading rod. The above procedures were mainly performed to increase the stability of the junction between the loading rod and the loading point.

### Preparation for testing

The hemi-pelvis and fixture were fixed on a horizontal platform that could be moved in four directions. By moving the position of the platform, the loading point could match the ball head of the loading rod. Considering the poor stability of the SIJ in the hemi-pelvic state, a steel cable was placed at the pubic symphysis and fixed to a stationary platform. In addition, a force sensor was attached to the cable to monitor the tension on the pubic symphysis. Optical motion tracking markers were placed on both sides of the SIJ (Fig. 2B). Finally, the motion data from “O” point on the SIJ (the intersection of the arcuate line and SIJ) were captured by NDI Optotrak Certus (Fig. 2C and D).

### Biomechanical test

First, MOP tests were performed with the intact SIJ specimen, and then SIJ stability tests were performed.

MOP tests: MOP-F1 (F: force) and F2 were randomly and sequentially applied to the nine specimens. In MOP-F1, the direction of loading force was at a degree of 30 to the sagittal plane of the pelvis. In MOP-F2, the direction of the loading force was parallel to the sagittal plane of the pelvis (Fig. 2E and F).

The detailed procedures were as follows:

- The procedure of MOP-F1: The hemi-pelvis was placed at a degree of 30 to the sagittal plane of the pelvis. The ball head was combined with loading point 2. A force of 10 N was preapplied to make the ball head fully contact the loading point. Then, a vertical downwards load of 10 ~ 100 N was applied with a loading frequency of 0.025 Hz. After the loading force reached 100 N, it was gradually unloaded to 10 N. Then, the next cycle was repeated. The loading was cyclic and performed three times for each specimen, and the data from the third cycle were taken as experimental data.
- The procedure of MOP-F2: The hemi-pelvis was placed in a supine position. The ball head was combined with loading point 1. The loading mode was the same as that of MOP-F1.
- SIJ stability tests: After the MOP-F1 and F2 tests, the tests of stability of the SIJ were continued. The ASL was divided into two parts (upper and lower) by the intersection of the arcuate line and the SIJ. The six hemi-pelvises were randomly divided into two groups. In Group 1, the upper part of the ASL was resected, followed by the lower part. In Group 2, the lower part of the ASL was resected, followed by the upper part. The loading mode was the same as MOP-F2.

### Analysis

Statistical analyses were performed using SPSS version 20.0 software (IBM SPSS Statistics for Windows, Armonk, NY, USA). Data are presented as averages ± standard deviations. The paired t test was used to evaluate the differences in the rotation angle and displacement of the SIJ between MOP-F1 and F2. In addition, the paired t test was used to evaluate the contributions of each part of ASL within each group [23]. The paired t test was used to evaluate the differences in the rotation angle and displacement of the SIJ between the states of intact ASL and resected ASL. The Mann–Whitney U test was used to compare the two parts of ASL contributions between the two groups [23]. A *p* value < 0.05 indicated a statistically significant difference.

## Results

### Rotation angles of intact SIJ under MOP-F1 and F2

**Fig. 3:**
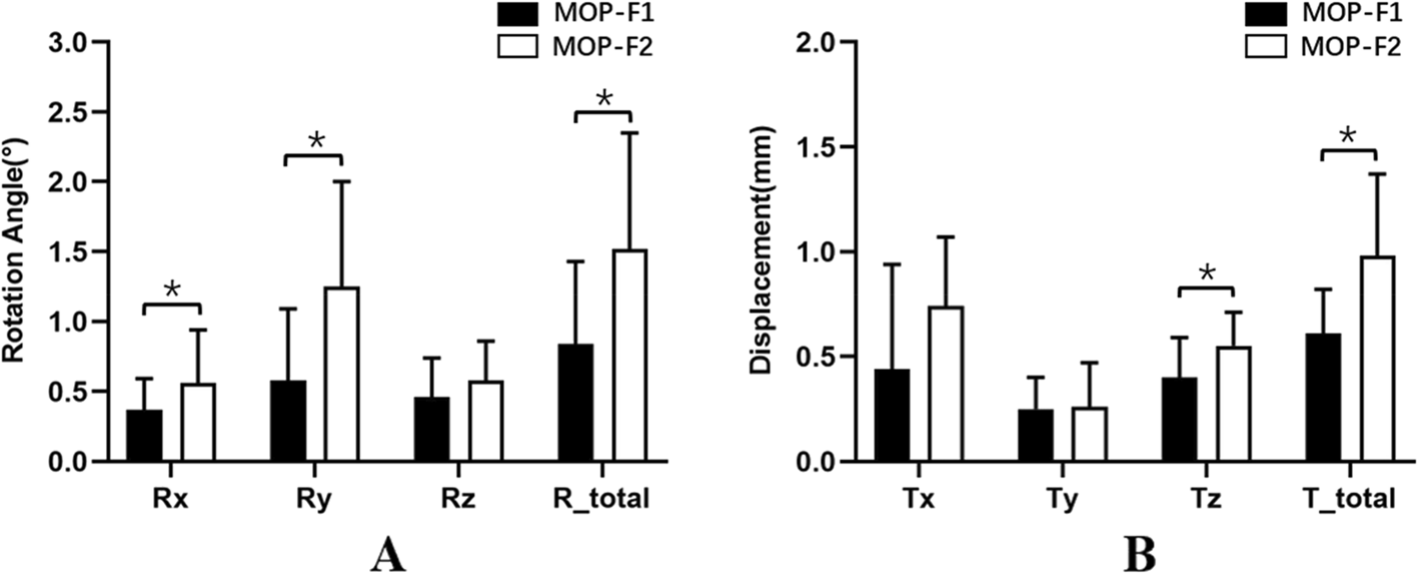
**A** and **B** showed that the MOP-F1 and F2 applying to hemi-pelvises produced the rotation angle and displacements of SIJ, respectively. Rx represented the angle of rotation on a coronal axis. Ry represented the angle of rotation on a vertical axis. Rz represented the angle of rotation on a sagittal axis. R_total represented the total rotation angle. Tx represented the displacement on a coronal axis. Ty represented the displacement on a vertical axis. Tz represented the displacement on a sagittal axis. T_total represented the total displacement. The symbol “*” indicated that there was a statistical difference between two groups, that was, p<0.05. MOP: Manipulation of oblique pulling. SIJ: Sacroiliac joint

The rotation angle of the SIJ surface gradually increased with increasing loading force under the two MOPs. Under MOP-F1 and F2, the average total angles were 0.84° ± 0.59° and 1.52° ± 0.83°, respectively. There was a significant difference between the two MOPs for total angle (t = − 6.34, *p* = 0.00). The average rotation angle on the vertical axis of the pelvis was the largest among the three directions. Compared with MOP-F1, MOP-F2 caused greater rotation angles of the SIJ surface on the coronal and vertical axes, and the differences were statistically significant (t = − 2.46, *p* = 0.04; t = − 5.27, *p* = 0.00) (Fig. 3A). The average angles in each direction are summarized in Table 1.

**Table 1:** The data of average rotation angles and displacements of SIJ produced by MOP-F1 and F2

	MOP-F1	MOP-F2
Rx (°)	0.37 ± 0.22	0.56 ± 0.38
Ry (°)	0.58 ± 0.51	1.25 ± 0.75
Rz (°)	0.46 ± 0.28	0.58 ± 0.28
Tx (mm)	0.44 ± 0.50	0.74 ± 0.33
Ty (mm)	0.25 ± 0.15	0.26 ± 0.21
Tz (mm)	0.40 ± 0.19	0.55 ± 0.16
R_total (°)	0.84 ± 0.59	1.52 ± 0.83
T_total (mm)	0.61 ± 0.21	0.98 ± 0.39

Rx represents the rotation angle on a coronal axis. Ry represents the rotation angle on a vertical axis. Rz represents the rotation angle on a sagittal axis. Tx represents the displacement on a coronal axis. Ty represents the displacement on a vertical axis. Tz represents the displacement on a sagittal axis. R_total represents the total rotation angle. T_total represents the total displacement. SIJ: Sacroiliac joint. MOP: Manipulation of oblique pulling

### Displacements of intact SIJ under MOP-F1 and F2

The displacement of the SIJ surface gradually increased with increasing loading force under the two MOPs. Under MOP-F1 and F2, the average total displacements were (0.61 ± 0.21) mm and (0.98 ± 0.39) mm, respectively. There was a significant difference between the two MOPs for total displacement (t = − 3.87, *p* = 0.01). The average displacement on the coronal axis of the pelvis was the largest among the three directions. Compared with MOP-F1, MOP-F2 caused a greater displacement on the sagittal axis, and the difference was statistically significant (t = − 4.13, *p* = 0.01) (Fig. 3B). The displacements in each direction are summarized in Table 1.

### Effect of ASL

**Fig. 4:**
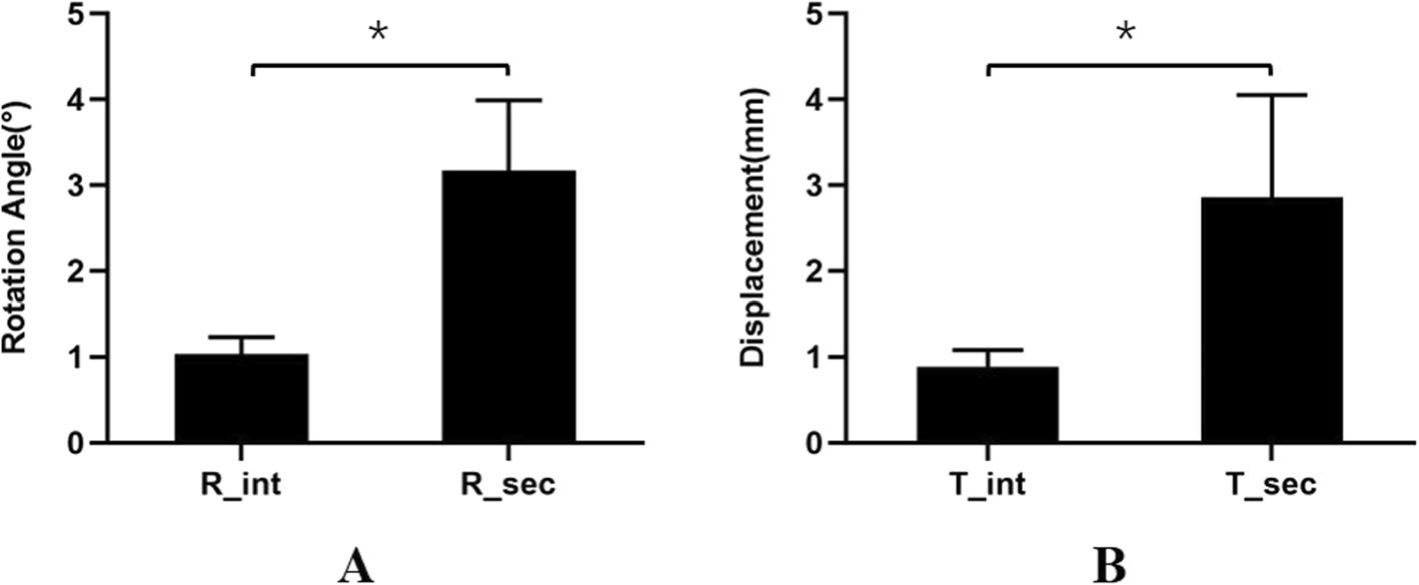
**A** and **B** showed that the rotation angle and displacement of SIJ in the states of intact ASL and ASL resection, respectively. R_int and T_int represented the rotation angle and displacement in the state of intact ASL. R_sec and T_sec represented the rotation angle and displacement in the state of ASL resection. The symbol “*” indicated that there was a statistical difference between the two states, that was, *p *< 0.05. SIJ: Sacroiliac joint. ASL: Anterior sacroiliac ligament

Six specimens were included in the SIJ stability test. In the intact ASL and resected ASL states, the rotation angles of the SIJ were 1.03° ± 0.20° and 3.17° ± 0.82°, and the displacements of the SIJ were 0.88 ± 0.20 mm and 2.86 ± 1.19 mm, respectively. Both the rotation angle and displacement of the SIJ in the state of resected ASL were significantly larger than those of the intact ASL (t = − 5.30, *p* = 0.01; t = − 4.08, *p* = 0.02) (Fig. 4A and B).

### Effect of the two parts of ASL

**Fig. 5:**
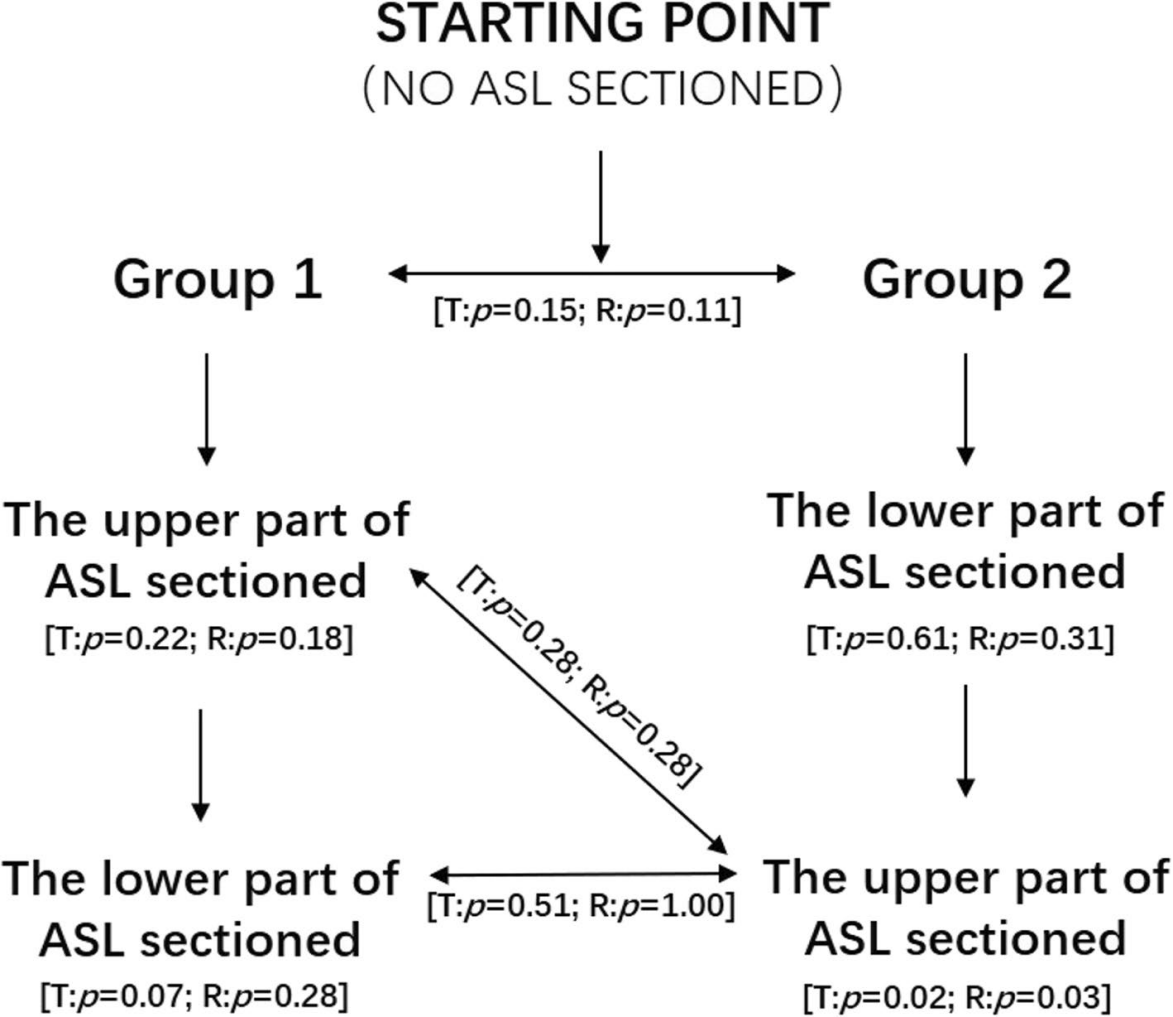
Flow diagram showing the study design with the associated statistical result at each stage of ASL resection of two parts. There was no significant change within groups after sectioning of the lower part of ASL whether this was performed before or after the upper part of ASL sectioning. In addition, there was no significantly difference between groups after sectioning of the upper part of ASL whether this was performed before or after sectioning of the lower part of ASL. “T” represented displacement. “R” represented rotation angle. ASL: Anterior sacroiliac ligament

In Group 1, in the intact state, the upper part resection and complete resection of ASL, the rotation angles of the SIJ were 1.45° ± 0.36°, 2.47° ± 1.22° and 3.01° ± 0.45°, and the displacements were 0.99 ± 0.19 mm, 1.83 ± 0.96 mm and 3.30 ± 1.45 mm, respectively. The differences were not statistically significant among the three states for the rotation angle and displacement. In Group 2, in the intact state of the lower part resection and the complete resection of ASL, the rotation angles of the SIJ were 0.89° ± 0.06°, 1.50° ± 0.74° and 3.27° ± 1.10°, and the displacements were 0.72 ± 0.01 mm, 1.17 ± 0.64 mm and 2.73 ± 0.93 mm, respectively. There were significant differences between the states of lower part resection and complete resection for the rotation angle and displacement (t = − 5.69, *p* = 0.03; t = − 7.06, *p* = 0.02) (Fig. 5).

## Discussion

SIJ dysfunction interferes greatly with people’s work and life. Manipulation has the advantages of minimal invasiveness and good curative effects, and it is widely used in clinical practice. However, the biomechanical mechanism of manipulation remains unclear. Clarifying its mechanism helps to improve the effect of manipulation, which is of great significance to the treatment of SIJ disorders.

Miller et al. studied the correlation between the loading force and displacement of the SIJ using eight specimens. By fixing the bilateral ilium, they applied 294 N of force from the upper, lower, anterior, posterior, and middle sides and 42 Nm torque of flexion, extension, lateral flexion, and rotation to the sacrum. They found that the average SIJ displacements were 0.28 mm, 0.26 mm, 0.48 mm, 0.53 mm and 0.01 mm, and the average rotation angles were 1.31°, 1.94°, 0.37° and 0.80°, respectively [24]. Lindsey and colleagues performed a cadaveric study to investigate the motion of the SIJ and found that under the loading conditions of flexion-extension, rotation and lateral flexion, the ROMs of the SIJ were 1.94° and 2.65°, 1.20° and 1.77°, and 0.36° and 1.16° in the single- and double-leg stance states, respectively [25]. In this experiment, nine hemi-pelvic specimens were used to study the effects of MOPs in different directions on the SIJ. The results showed that under a 100 N loading force, the total rotation angles of F1 and F2 were 0.84° and 1.52°, and the displacements were 0.61 mm and 0.98 mm, respectively. The ROM of the SIJ was consistent with previous studies [24, 25]. MOP-F2 could produce a greater rotation angle and displacement of the SIJ, which might be related to the fact that the force direction of MOP-F2 was more perpendicular to the SIJ surface, and the torque was greater. The rotation angles on the vertical axis produced by MOP-F1 and F2 were the largest among the three directions. MOPs were applied to the anterior superior iliac spine. The loading force applied from the ventral to the dorsal side caused the ilium to rotate outwardly with respect to the sacrum, so the rotation angle was at its maximum on the vertical axis. In the three directions, the displacements on the coronal axis caused by MOP-F1 and F2 were the largest, while the displacements on the vertical axis were the smallest. The results suggested that the main effect of MOP was to produce coronal axis movement of the SIJ. In brief, MOP can cause movement of the SIJ, but the ROM is small. MOP-F2 is a more effective manipulation.

The ligaments perform a vital role in holding the different components of the structure together against loads, which otherwise would cause separation at the pubis and SIJs. Sichting et al. considered that ligaments served as the mechanical stabilization device of the pelvis [18]. Pool-Goudzwaard et al. found that when rotational torque was applied to the SIJ in the sagittal plane, the iliolumbar ligament could obviously constrain the SIJ motion, and the ventral band had the greatest effect on SIJ motion [26]. Eichenseer et al. indicated that the ligaments around the SIJ could restrict its movement and decrease its stress through a finite element study [22]. Abdelfattah and colleagues found that the pubic symphysis and the ASL played a greater role in maintaining the stability of the pelvis when the pelvis suffered an open book injury [23]. In this study, it was found that after complete resection of the ASL, the displacement of the SIJ increased by 243%, and the rotation angle increased by 171%. It also proved that the ASL played an important role in maintaining the stability of the SIJ.

The sacrum is wedge-shaped, tilted from top to bottom and has a concave surface that is closely inserted into the convex surface of the ilium [27]. Since humans are upright, the lower part of the SIJ surface fits more tightly. In addition, a previous CT imaging study showed that the SIJ space width gradually narrows from top to bottom [28]. Theoretically, the SIJ gap is wider, the ROM of the SIJ is larger, and the ligaments maintaining SIJ stability bear greater strains. The results showed that, compared with the intact state of ASL, resecting the upper part increased the rotation angle and displacement of the SIJ by 70 and 85%, while resecting the lower part increased the rotation angle and displacement of the SIJ by 69 and 63%, respectively. After resecting the upper part, the rotation angle and displacement of the SIJ increased by 22 and 80%, respectively, by resecting the lower part. After resecting the lower part, the rotation angle and displacement of the SIJ increased by 118 and 133%, respectively, by resecting the upper part. The data indicated that the upper part of the ASL played a more important role in maintaining the stability of the SIJ than the lower part.

There are some limitations of this study. First, the pelvic specimens were hemi-pelvises, and the symphysis pubes had been dissected, which affected the stability of the SIJ. To this end, we took the following measures: (1) A smaller loading force of 100 N was applied; (2) A steel cable was placed at the symphysis pubis to restrict the movement of the symphysis pubis, and the tension was monitored. The forces caused by MOP-F1 and F2 were 7.62 N and 13.85 N, approximately 10% of the loading force. The results indirectly reflected that the movement of the symphysis pubis was small. Thus, the hemi-pelvis specimens had little effect on the experiment. Second, only six specimens were included in the ASL experiments, so the number of specimens was small. Finally, fresh pelvis specimens were studied, and the intact surrounding ligaments of the SIJ were preserved. However, the simulative MOP in this study could not fully reflect the characteristics of clinical MOP in vivo.

## Conclusions

This novel study was the first to investigate the biomechanical mechanism of MOP on the SIJ with human pelvis specimens and clarify the effect of different loading directions of MOP on the movement of the SIJ. In addition, this study confirmed that the ASL plays an important role in maintaining the stability of the SIJ and suggested that the upper part of the ASL contributes more than the lower part to SIJ stability.

## Data Availability

The data used and/or analyzed during the current study are available from the corresponding author on reasonable request.

## Abbreviations

SIJ: Sacroiliac joint
MOP: Manipulation of oblique pulling
ASL: Anterior sacroiliac ligament
LPSL: Long posterior sacroiliac ligament
SPSL: Short posterior sacroiliac ligament
ISL: Interosseous sacroiliac ligament
SS: Sacrospinous ligament
ST: Sacrotuberous ligament
ASIS: Anterior superior iliac spine
ROM: Range of motion
